# MEDICATION DE-ESCALATION AND GLYCATED HEMOGLOBIN AMONG OLDER ADULTS WITH DIABETES AND DEMENTIA

**DOI:** 10.1093/geroni/igad104.2258

**Published:** 2023-12-21

**Authors:** Oluwaseun Adeyemi, Mauricio Arcila-Mesa, Crystalinda Rapozo, Joshua Chodosh

**Affiliations:** New York University Grossman School of Medicine, New York City, New York, United States; NYU Langone, New York City, New York, United States; NYU Grossman School of Medicine, New York City, New York, United States; NYU Grossman School of Medicine, New York City, New York, United States

## Abstract

Patients with co-occurring diabetes and dementia and low (< 7.0) glycated hemoglobin (HbA1c) are at risk for adverse events and may require antihyperglycemic medication de-escalation. Whether de-escalation leads to a desired HbA1c is unknown. We assessed the relationship between antihyperglycemic de-escalation and attainment of a desired HbA1c. We selected older adults with diabetes and dementia with low HbA1c enrolled in the Enhanced Quality in Primary Care for Elders with Diabetes and Dementia, a multicenter cluster-randomized pragmatic trial. We determined whether antihyperglycemic medications were de-escalated during the twenty-four-month intervention. We classified a positive outcome as a subsequent desired HbA1c range for those who entered the study with HbA1cs < 7.0. We controlled for age, sex, race/ethnicity, Charlson comorbidity index, antihyperglycemic medications, and the COVID-19 pandemic. We performed a multivariable generalized linear binomial regression analysis and report the likelihood (adjusted risk ratio; aRR) (plus 95% confidence interval (CI)) of achieving the desired range HbA1c. The predominant population was older adults 85 years and older (42%), females (60%), and non-Hispanic Whites (64%). A tenth of the population had de-escalation and 19% of the population achieved the desired HbA1c range at the end of the study period. Metformin was the most frequently retained medication after de-escalation (66.7%). After adjusting for potential confounders, de-escalation was associated with 2.2 times the likelihood of attaining the desired HbA1c range (aRR: 2.20; 95% CI: 1.34 – 3.55). In conclusion, antihyperglycemic medication de-escalation improves the glycemic control of older adults with DM/ADRD.

